# Comparing explicit and implicit ensemble perception: 3 stimulus variables and 3 presentation modes

**DOI:** 10.3758/s13414-023-02784-4

**Published:** 2023-10-11

**Authors:** Noam Khayat, Marina Pavlovskaya, Shaul Hochstein

**Affiliations:** ELSC Safra Center for Brain Research and Life Sciences Institute, Hebrew University, Jerusalem, 91904 Israel

**Keywords:** Dual-task performance, Visual perception, Visual awareness

## Abstract

Visual scenes are too complex for one to immediately perceive all their details. As suggested by Gestalt psychologists, grouping similar scene elements and perceiving their summary statistics provides one shortcut for evaluating scene gist. Perceiving ensemble statistics overcomes processing, attention, and memory limits, facilitating higher-order scene understanding. Ensemble perception spans simple/complex dimensions (circle size, face emotion), including various statistics (mean, range), and inherently spans space and/or time, when sets are presented scattered across the visual scene, and/or sequentially in rapid series. Furthermore, ensemble perception occurs explicitly, when observers are asked to judge set mean, and also automatically/implicitly, when observers are engaged in an orthogonal task. We now study relationships among these ensemble-perception phenomena, testing explicit and implicit ensemble perception; for sets varying in circle size, line orientation, or disc brightness; and with spatial, temporal or spatio-temporal presentation. Following ensemble set presentation, observers were asked if a test image, or which of two test images, had been present in the set. Confirming previous results, responses reflected implicit mean perception, depending on test image distance from the mean, and on its being within or outside ensemble range. Subsequent experiments asked the same observers to explicitly judge whether test images were larger, more clockwise, or brighter than the set mean, or which of two test images was closer to the mean. Comparing implicit and explicit mean perception, we find that explicit ensemble averaging is more precise than implicit mean perception—for each ensemble variable and presentation mode. Implications are discussed regarding possible separate mechanisms for explicit versus implicit ensemble perception.

## Introduction

Gestalt psychologists suggested that similar scene elements are grouped (Koffka, 1935; Wagemans et al., 2012; Wertheimer, 1923/1938) so that perception of the group’s spatial arrangement and its summary statistics provide a shortcut toward evaluating the gist of complex scenes (Ariely, 2001; Cohen et al., 2016; Hochstein & Ahissar, 2002). In fact, the idea of set representation was long known, including the phenomenon of central tendency or regression to the mean. For example, Hollingworth (1910) noted that magnitude estimates tend to gravitate towards a value equal to the mean of the set. It has been suggested that perceiving ensembles rather than individuals expands processing, attention, and memory limits (Alvarez, 2011; Cohen et al., 2016; Utochkin, 2016).

Numerous studies since the turn of the millennium have found that we rapidly perceive set mean values for multiple object features, including size (Ariely, 2001; Bauer, 2015; Chong & Treisman, 2003; Corbett & Oriet, 2011), orientation (Dakin & Watt, 1997; Parkes et al., 2001), brightness (Bauer, 2009; Chetverikov et al., 2017; Takano & Kimura, 2020), color (Olkkonen et al., 2014; Webster et al., 2014), position (Alvarez & Oliva, 2008; Lew & Vul, 2015), and face identity, gender, emotional expression, eye-gaze, or general lifelikeness (de Fockert & Wolfenstein, 2009; Haberman & Whitney, 2007, 2009; Sweeny & Whitney, 2014; Yamanashi Leib et al., 2014). Perceived statistics also include set feature variance or range (Dakin & Watt, 1997; Haberman & Whitney, 2012; Pollard, 1984), in the visual and auditory domains (McDermott et al., 2013; Schweickert et al., 2014) and separate statistics for separable sets of elements (Chong & Treisman, 2003; Haberman & Whitney, 2012). While set statistics may affect perception, memory, and/or decision-making, we follow all the above, calling the phenomenon “ensemble perception.” For ensemble perception reviews, see Haberman and Whitney (2012), Bauer (2015), Cohen et al. (2016), and Corbett et al. (2023).

**Fig. 1:**
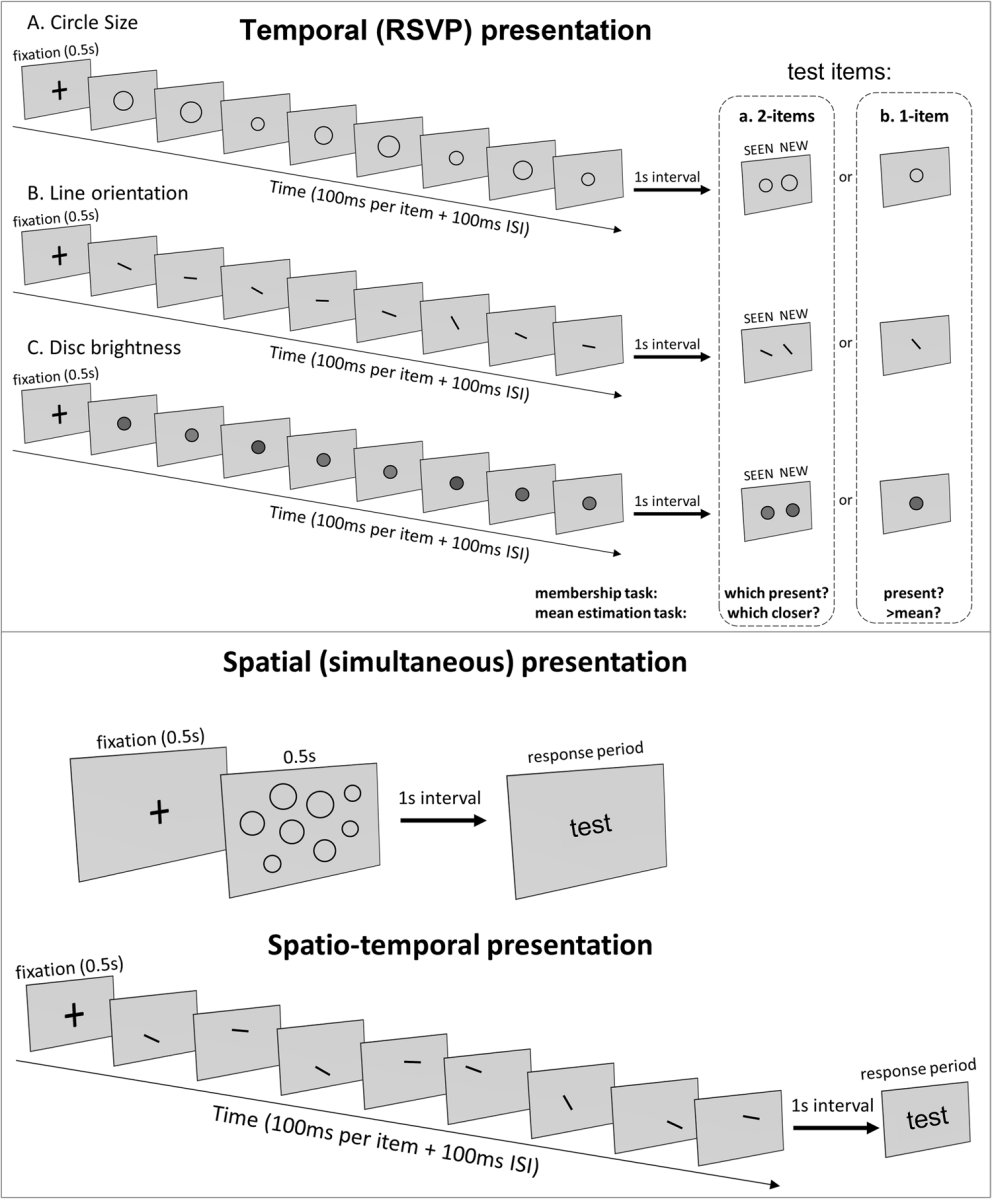
Top: Implicit and explicit ensemble perception tests. Rapid Serial Visual Presentation (RSVP) of a sequence of images differing in **A** circle size, **B** line orientation, or **C** disc brightness is followed by presentation of two test images (**a**). To test implicit ensemble perception, observers are asked which image was present in the sequence. Their responding according to the shorter distance from the sequence mean indicates implicit set mean perception. Explicit mean perception is tested by directly asking which of the two test images is closer to the mean. An alternative testing method (**b**) presents only a single test image and asks if it was present in the sequence (implicit mean perception) or if it is larger, more clockwise, or brighter than the set mean (explicit mean perception). Bottom: illustration of the other presentation modes—spatial (e.g., circle size) and spatiotemporal (e.g., line orientation). These were followed by the same 2 or 1 test image(s) as in (**a**). See text

**Fig. 2:**
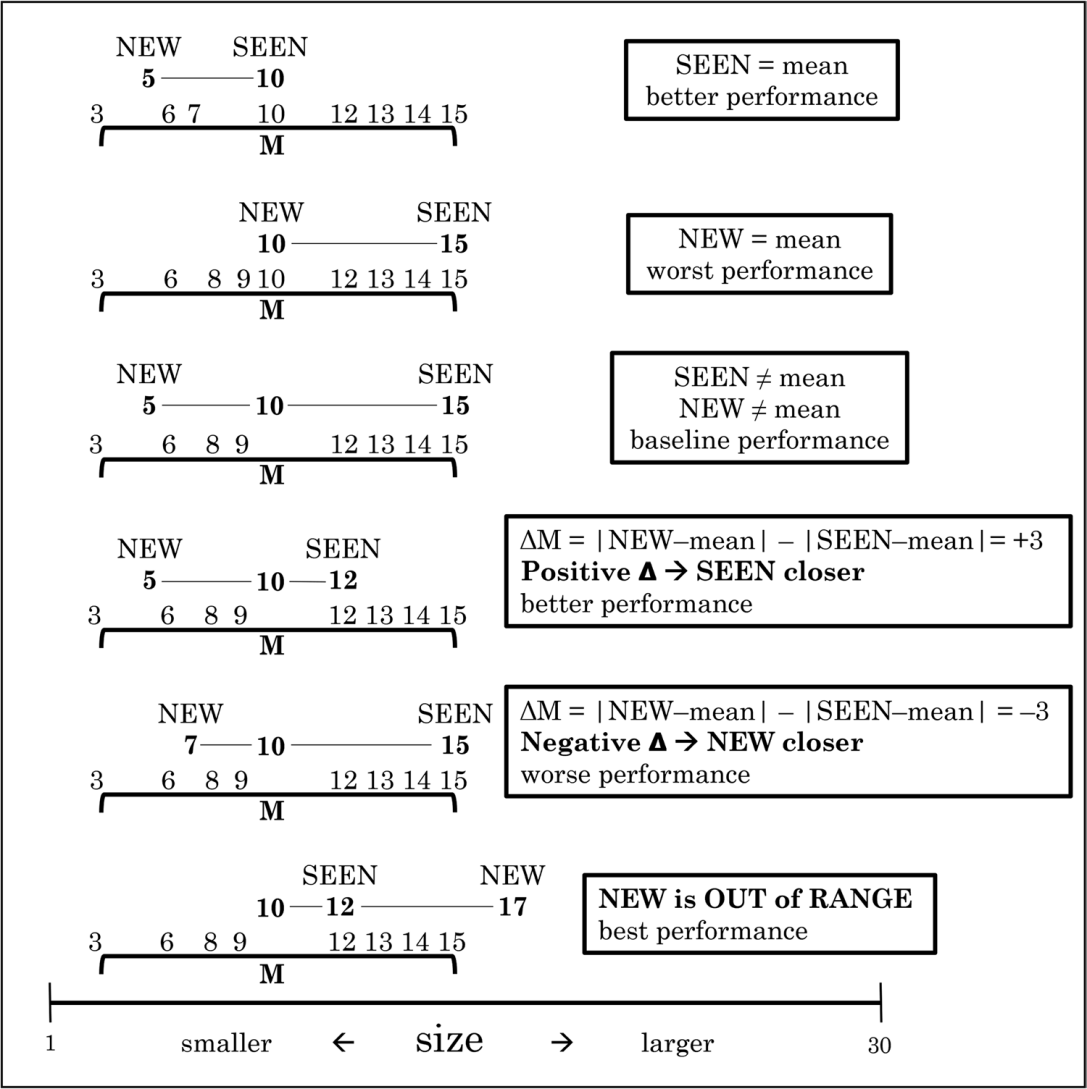
Examples of different *implicit-2-test-images* trial subtypes. In each case, the 8 sequence members are indicated, as well as their mean (M) and the two test images (SEEN and NEW). From the top: SEEN test image = sequence mean, expecting observers to correctly choose it; NEW = mean, expecting incorrect choice of NEW image; neither = mean, SEEN and NEW equidistant from mean, expecting ~50% chance performance; SEEN closer, better than 50%; NEW closer, less than 50%; NEW out of range, expecting easy rejection of NEW, and choice of SEEN, whether = mean or not. Note that performance accuracy is measured by choice of the SEEN test image and not by of the test image closer to the mean, though our central interest is the effect of mean perception on this choice

Nearly all of the above experiments tested *explicit* perception of the ensemble mean—that is, participants were asked to evaluate the mean and perform a task related to this mean. Explicit perception is deliberate and conscious, cognitively demanding with top-down attention (Cohen et al., 2016; Hochstein & Ahissar, 2002; Reber et al., 1999). On the other hand, Khayat and Hochstein (2018, 2019; Hochstein, 2020; Khayat et al., 2021) studied *implicit* perception and memory of set statistics (see also Hansmann-Roth et al., 2021). Implicit perception is automatic and nonconscious, believed to involve bottom-up sensory integration (Cohen et al., 2016; Hochstein & Ahissar, 2002; Reber et al., 1999). Khayat, Fusi, and Hochstein (2021) presented a rapid serial visual presentation (RSVP) sequence of images differing by low-level properties (circles of different size, lines of different orientation, discs of different brightness; see Fig. 1, Top, a), and tested only memory of membership in the sequence of test images or items. The mean of the set—mean size circle, mean orientation line, or mean brightness disc—was sometimes included in the set sequence, and sometimes absent. After showing the set RSVP, they presented two images, side by side, simultaneously, one SEEN in the sequence and one not present, a NEW image. They tested observer perception and memory by asking participants to choose which test image had been SEEN in the sequence. They did not inform observers that one test element could be the sequence mean, whether the SEEN test image (i.e., a RSVP sequence member) or the NEW foil image (i.e., not a sequence member). Also, they did not inform them that sometimes the NEW test image was outside the sequence range. They purposely did not mention in their instructions the words “mean” and “range,” in order to test if observers automatically perceive set mean and choose test images that match, or are closer to the mean. They also asked if observers would automatically perceive set property range and easily reject foils outside the sequence range. These test-stimulus contingencies, called “trial subtypes,” are shown in Table 1, and demonstrated in Fig. 2, using the terms: “in” and “out”—test elements within and outside the range of the variable sequence property; “mean”—element with property equal to sequence mean. Baseline performance is for the subtype where neither test item equals the mean, and they are, on average, equidistant from the mean. Note that “performance” accuracy is measured by choice of the SEEN test image and not by choice of the test image closer to the mean, even though we are interested in the effect of mean perception on this choice. Thus, choice of the NEW test image, when it equals the set mean, is deemed incorrect (leading to poor performance) in terms of memory of the set, and at the same time reflects (misleading) perception of the mean.

**Table 1:** Trial subtypes and expected performance in the membership task (see also Fig. 2)

SEEN member test image (correct)	NEW member test image (incorrect)	expected performance
in range	out of range	best
mean	in range	better
in range	in range	baseline
in range	mean	worse

Khayat and Hochstein (2018) found that when participants choose which of two test stimuli was present in the preceding RSVP sequence, they tend to select the test image that is closer to the set mean property, even when it was never presented, suggesting that average size, orientation, and brightness are automatically and implicitly encoded (see Maule et al., 2014, regarding mean color). Note that the variables tested, size, orientation and brightness, have different representations in visual cortex (Gardner et al., 2005; Konkle & Oliva, 2012; Shapley et al., 2003). These findings confirmed earlier results by Corbett and Oriet (2011) who used an attentional blink paradigm and found implicit perception of an RSVP sequence mean. Similar characteristics were also found when testing memory of objects belonging to a particular category, when presented in RSVP sequence, with observers perceiving the objects’ category prototype (similar to set mean) and the category itself (similar to range; Khayat et al., 2021; Khayat & Hochstein, 2019).

In the current study, we test both implicit and explicit ensemble perception, comparing their precision in the same participants. Furthermore, while most studies presented ensemble stimuli simultaneously, and only a few presented them serially (e.g., Corbett & Oriet, 2011; Khayat & Hochstein, 2018), we now test three presentation modes (temporal, spatial and spatio-temporal) allowing direct comparison of results reflecting integration mechanisms over time and/or space. Thus, a central goal of the present study is comparison of ensemble perception of different features, different presentation, and explicit/implicit processing.

The widespread parallels at multiple levels of cortical representation suggest that they reflect basic brain-processing principles. Here we also seek to determine the relationships among the mechanisms underlying these different tasks. Is there a single “averaging” mechanism that performs mean perception for sets differing in various features, and/or spread over space or time, and when observers perform an averaging task or implicitly perceive the mean when engaged in an unrelated task, or are there separate cerebral mechanisms for some or all of these different tasks?

Previous studies have investigated the relationships among ensemble perception of different features with mixed results. Comparing performance of two low-level features (length and orientation of lines) yielded mixed results with significant (Kacin et al., 2021) and nonsignificant (Yörük & Boduroglu, 2020) individual-differences correlations. Tests of high-level object ensemble perception (planes, birds, cars) found significant correlations (Chang and Gauthier, 2021). On the other hand, Haberman et al. (2015) compared several low- and high-level stimulus ensemble representations and found no significant correlations. Taken together, these studies suggest there is no “domain-general” mechanism, though there may be common mechanisms for similar level features. Note that even if the same computation is used for different features, it may be performed by repeated, local mechanisms, for each.

Besides the two-test image paradigm described above and in Fig. 1, Top, a, in the current study we also use an alternative testing paradigm, with a single test image, as shown in Fig. 1, Top, b. For the implicit ensemble test, participants are asked if the test image was a member of the set; for the explicit test, they are asked if it is greater than the set mean (larger size, more clockwise orientation, or lighter brightness). Similar to the 2-test image paradigm, the single test image could be a sequence member (SEEN) or not included in the sequence (NEW), could equal the mean (if SEEN, better implicit membership-task performance, if NEW, worse implicit membership-task performance), or, if NEW, could be outside the sequence range (best implicit membership-task performance by easy rejection, and best explicit performance by easy comparison to perceived mean). The importance of using these two testing paradigms is in more direct comparison of two tests of implicit mean perception with 2 test images, and more direct comparison of explicit and implicit mean perception with a single test image.

## Methods

### Participants

Ninety-six master workers were recruited from the Amazon Mechanical Turk (MTurk) platform, a crowdsourcing platform enabling coordination of online participants of uploaded human information tasks. Each observer participated in 6 experimental sessions, 3 testing implicit ensemble perception, followed by 3 testing explicit mean perception. In each case, the 3 sessions differed in presentation mode, one each with temporal, spatio-temporal, or spatial presentation, in this order. Each session had 3 blocks of trials testing circle size, line orientation and disc brightness, respectively, in this order. 96 participants completed the full 6 experimental sessions (55 the *2-test image* paradigm; 41 the *1-test image* paradigm).

### Stimuli

All stimuli were created using python 3.7, and the experiment was designed using JavaScript and uploaded to the online MTurk platform. Stimuli in the different blocks of each experimental session were either circles with different sizes, bars with different orientations, or discs with different brightness. Each set contained 8 images, presented in random order and/or position. The distributions of stimuli were as follows. The full range of stimuli was divided into 30 equidistant arbitrary units; for each trial, the range was limited to 8-21 units, and the difference between adjacent-size stimuli in a trial was below 5 units.

In circle-size blocks, ensembles consisted of hollow circles with different diameters. Each arbitrary unit of size represents an incremental radius of six pixels (for spatial presentation, five), so the full range of sizes was 1-30 units or 6-180 pixels (for spatial presentation, 5–150). In line-orientation blocks, each unit represents 6° and the full range of orientations was 6-180°. In disc-brightness blocks, each unit represents 2% of maximal screen brightness, and the full range of brightness was 21–79% of maximum screen brightness (RGB [256, 256, 256]). Disc diameter was 250 pixels, with a 5-pixel black border.

There were 100 trials per block (300 trials per session). In the *2-test image* sessions, the 100 trials included 40 baseline trials and 20 trials each for the other trial subtypes (SEEN = mean, NEW = mean, NEW = out). In the *1-test image* sessions, the 100 trials included 20 trials each for 5 trial subtypes (test image: SEEN = mean, NEW = mean, SEEN ≠ mean, NEW ≠ mean, NEW out of range).

### Experimental design

We used the implicit ensemble averaging paradigm, devised by Khayat and Hochstein (2018) and demonstrated in Fig. 1. Trial design was similar in all experiments, and the different conditions were determined by the test images' feature and presentation mode. Participants were instructed to sit 57 cm from a computer screen. Each trial began with a fixation cross appearing in the center of the screen for 500 ms and then, following observer press of the space bar, a set of 8 stimuli was presented by one of three modes: *temporal presentation*: serial sequence with 100 ms/stimulus and 100 ms interstimulus interval (ISI), followed by a masking stimulus to limit within-trial recency effects; *spatial presentation*: all 8 stimuli presented simultaneously for 500 ms positioned randomly within a 4 wide × 3 high lattice; *spatio-temporal presentation*: serial sequence of 100 ms/stimulus and 100 ms ISI, with stimuli in different positions (like those of the spatial presentation), in random order.

After presentation of the set stimuli, we had 4 test paradigms, in a 2 × 2 design: implicit vs. explicit and 2 test images vs. 1 test image. For the *implicit-2-test images* paradigm, a two-alternative-forced choice (2-AFC) membership task was tested: two test images were presented side-by-side and participants were instructed to indicate which one was a member of the sequence by pressing the keyboard's left or right arrow (Fig. 1a). Response time was unlimited, but we discarded response times that were longer than 3s. There was always one test image which was present in the set, i.e., the "SEEN" image (the correct response), and another one which was not—that is, the "NEW" image (the incorrect response). SEEN and NEW were pseudorandomly located on the left or right side of the display. For the *implicit-1-test image* paradigm, a single test image was presented in the middle of the screen; the image was randomly either SEEN or NEW, and participants were asked to judge if it had been presented in the set sequence. In either case, we expect a low chance of participants’ remembering which stimuli were in the set and which not. Instead, as we have found previously, they would base their decisions on the distance of the test stimuli from the set mean. It is in this way that these trials test implicit perception and memory of the set mean.

Only following 3 experimental sessions with implicit tests, either all with two or all with one test image, did we begin sessions with explicit mean tests. For the *explicit-2-test images* paradigm, participants were asked which of the two test images is closer to the set mean. For the *explicit-1-test image* paradigm, participants were asked if the test image was larger than the set mean circle size, more clockwise than the set mean line orientation, or brighter than the set mean disc brightness. Thus, in these sessions, we explicitly mentioned (for the first time) the notion of mean, and asked participants to assess the mean and use it for deciding on their responses.

The order of the Results section is as follows: First we present results of participants for whom we used the two test images paradigm, for implicit and then explicit tests, followed by their comparison. Next, we present results of participants for whom we used the one test image paradigm, again, for implicit and then explicit tests, followed by their comparison.

### Data analysis and statistical tests

The basic method of estimating the different implicit biases was a comparison of membership task accuracy for different trial conditions, assuming dependence on implicit mean perception. As already established using the *implicit-2-test image* paradigm (Khayat & Hochstein, 2018), we measured accuracy of determination of test image membership in the set for 4 different trial subtypes, as described in Fig. 2 and Table 1. Trial subtype was pseudo-randomly mixed in each session and participants were not aware of this division. To assess the gradual effect of test image distance from the mean, we measured test image membership performance as a function of the parameter Δ that represents the difference of the two test images’ distances from the mean (Fig. 2). Positive Δ corresponds to trials where the SEEN image is closer to the mean (increasing accuracy), and negative Δ corresponds to trials where the NEW image is closer to the mean (where we expect more frequent choice of the NEW test image, lowering accuracy to below 50%). This measure is more informative and detailed than the rough division to trial subtypes in which the test images are either exactly equal to the mean or not, as it incorporates the distances of both test images from the mean. This paradigm was found to provide robust effects of the trial mean and range on performance (Khayat & Hochstein, 2018, 2019; Khayat et al., 2021). The analysis of membership task performance versus Δ was also done separately for trials where both test images are within the trial range, to dissociate the mean effect from the robust range effect—that is, rejection of test images outside the set range.

Data were analyzed using MATLAB 2020b, SPSS 28.0 and Excel. Trials with RT below 200 ms or above 3 s were excluded from the analysis.

Membership trial results dependence on implicit mean perception was assessed by fitting a Gaussian curve to the data, following the equation: $y=a* {e}^{-\frac{{\left(x-c\right)}^{2}}{2{\upsigma }^{2}}}$, where *y* = fraction reporting “member” and *x* = distance of the (chosen) test image from the mean, with Gaussian parameters of height (a), width (σ), and center (c).

The gradual explicit mean effects by the distances of the test images from the mean was fit to the sigmoid function: $y=\mathit{min}+\frac{(max-min)}{1+{e}^{\left(-slope*\left(x-c\right)\right)}}$, where *y* = fraction reporting larger than or closer to the mean and *x* = distance of the 1 test image or difference of distances of the 2 test images from the mean, with sigmoid parameters of minimum (*min*), maximum (*max*), slope (*slope*), and center (*c*).

## Results

### Experiment 1— *2-test images-implicit* test paradigm

**Fig. 3:**
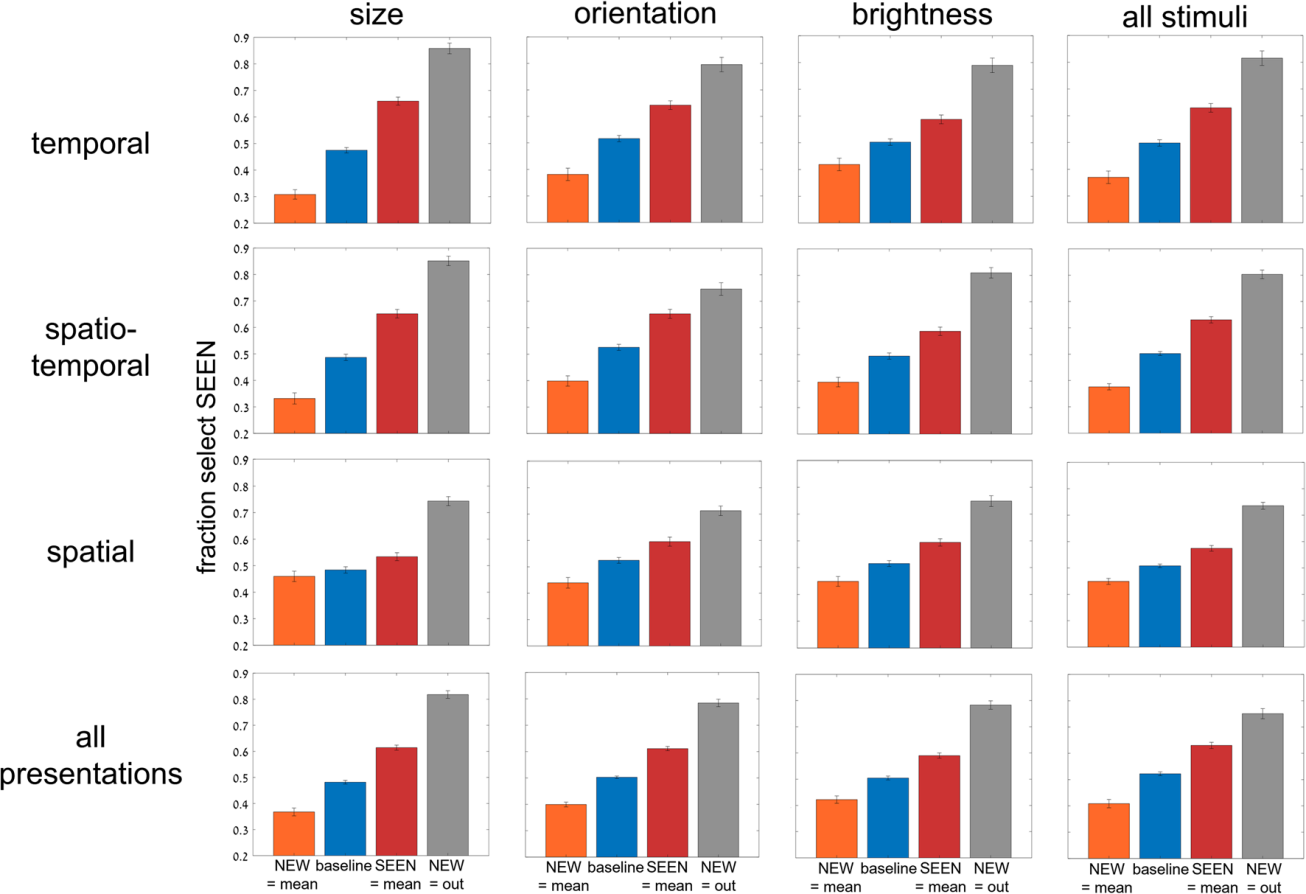
Experiment 1—*implicit, 2-test image* paradigm—Membership task performance as a function of trial subtype for three testing variables (columns: circle size, line orientation and disc brightness) and for three presentation modes (rows: temporal, spatio-temporal, and spatial). In every case, accuracy (proportion reporting that the SEEN image was present in the set) was greater for trials where SEEN = mean than those where NEW = mean, with the baseline subtype (neither = mean) between them, close to 50% chance performance. Best membership task performance was for NEW outside the sequence range and easily rejected. Error bars are standard error of the mean (*SEM*). (Color figure online)

**Fig. 4:**
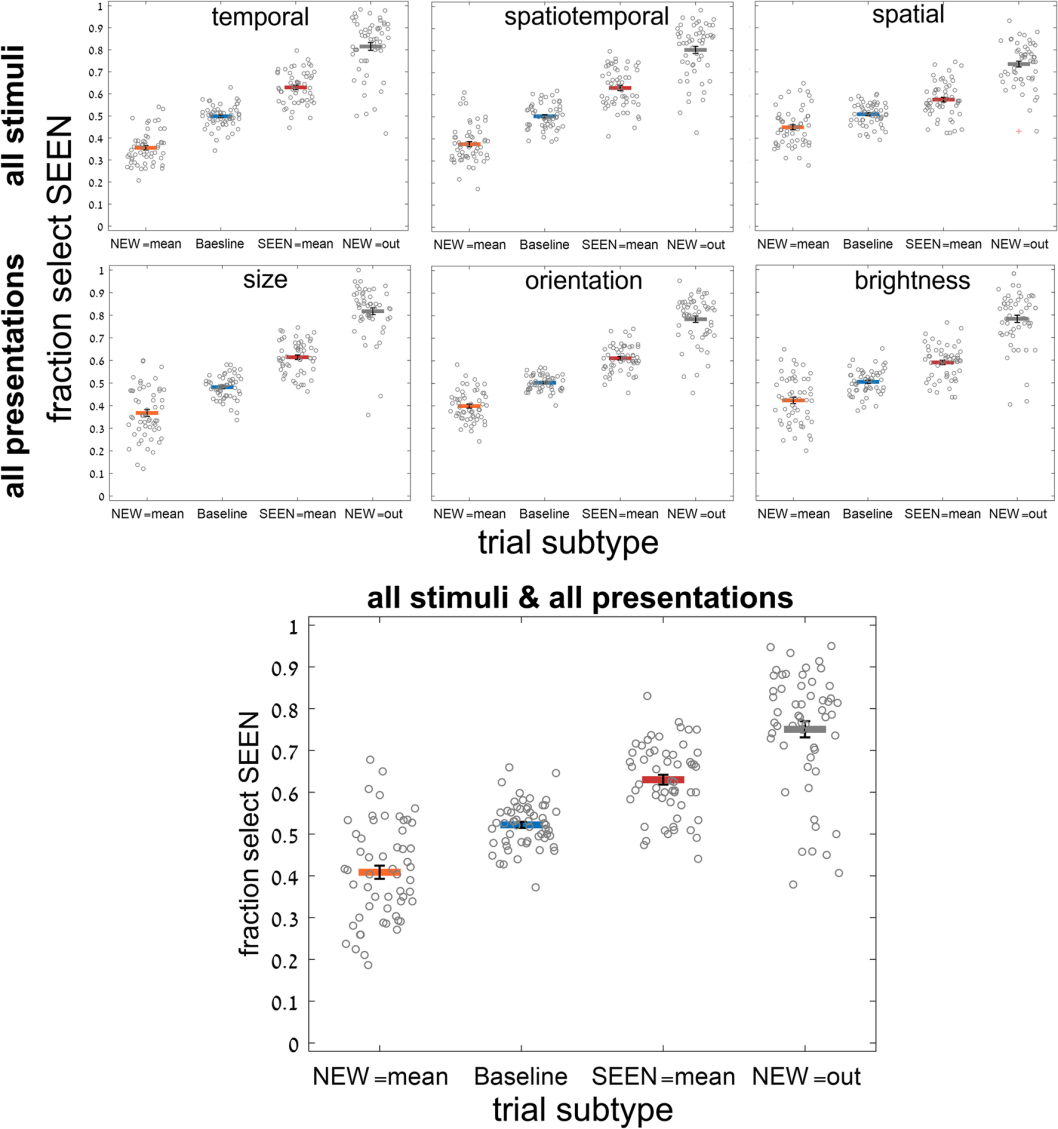
Experiment 1—*implicit, 2-test image* paradigm—Performance for individual participants as a function of trial subtype for 3 presentation modes, averaging over test variables (top), for 3 test variables, averaging over presentation modes (middle), and averaging over all cases (bottom). Despite considerable scatter among participants, average membership task performance is clearly, and significantly dependent on trial subtype. Each circle corresponds to a single participant’s performance; horizontal lines correspond to the average performance over participants; error bars are *SEM*. (Color figure online)

**Fig. 5:**
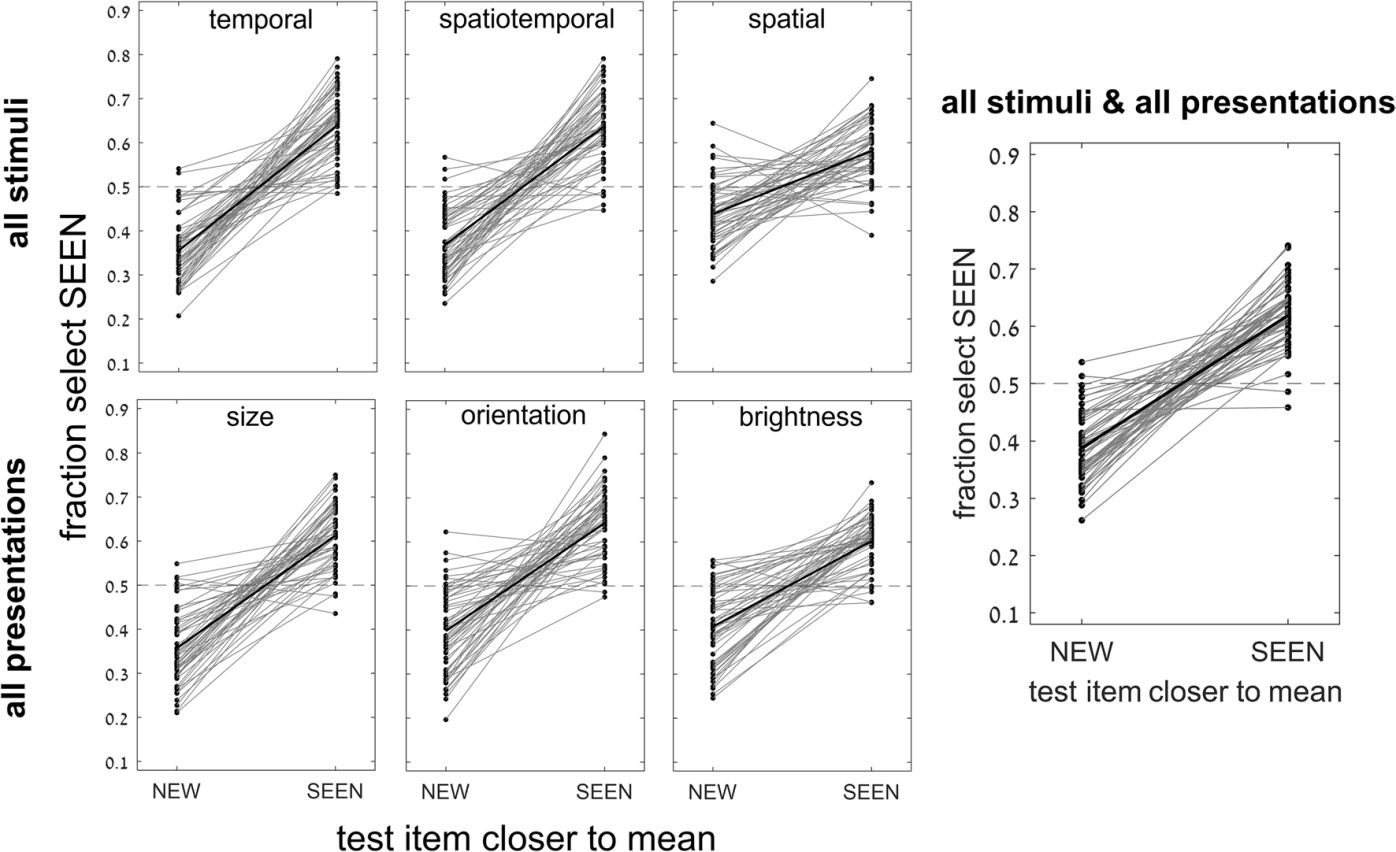
Experiment 1—*implicit, 2-test image* paradigm—Performance for individual participants as a function of which test image was closer to the mean, SEEN or NEW (excluding data for NEW out of set range). Performance, fraction choosing the SEEN image, was superior for almost all participants, in all conditions, when the SEEN image was closer to the mean, despite considerable scatter among participants. Each circle and line connecting performance for the two conditions, correspond to a single participant’s performance

We found consistent dependence on trial subtype, i.e., whether the SEEN or NEW test image was the set mean, neither was the set mean (baseline trials), or the NEW image was outside the set range; (see Fig. 2 and Table 1). As demonstrated in Fig. 3, participants tended to choose the SEEN or the NEW image when it was the set mean (the mean effect), even though the NEW image was never among the set images (red bars are above blue baseline reflecting choice of SEEN image when it equals the mean; orange bars are below baseline reflecting infrequent choice of SEEN image when the NEW image equals the mean). They were also better at choosing the SEEN image when they could reject the NEW image when it was outside the set range. These results were true, for all test variables (circle size, line orientation, disc brightness) and all presentation modes (temporal, spatio-temporal, spatial). Two-way repeated-measure ANOVA for subtype and presentation mode, with fraction choose SEEN as dependent variable, showed significant effects of subtype, *F*(3, 162) = 326, *p* < .001, and presentation, *F*(2, 108) = 3.3, *p* = .038, as well as a significant interaction between them, *F*(6, 324) = 19.6, *p* < .001, reflecting a smaller subtype dependence for spatial presentation. Two-way repeated-measure ANOVA for subtype and stimulus variable showed significant effect for subtype, *F*(3, 162) = 337, *p* < .001, but nonsignificant effects of stimulus variable, *F*(2, 108) = 0.795, *p* = 0.45; with, nevertheless, a significant interaction between them, *F*(6, 324) = 8.1, *p* < .001, due to a slightly reduced subtype dependence for brightness. The implicit effect of trial statistics (i.e., mean and range) assessed by comparing the different pairs of trial subtypes (SEEN = mean or NEW = mean vs. baseline, SEEN = mean vs. NEW = mean, and NEW = out vs. baseline), was highly significant and had large effect size for all presentation and stimulus variable blocks; for all comparisons, *p* < .001 and effect size Cohen’s *d* >0.7 (7/36 cases *d*>0.5), except for the case of spatial presentation, circle size, where *p* < .02 (*d* = 0.35) for SEEN = mean vs. NEW = mean or baseline, and *p* = 0.28 (*d* = 0.14) for NEW = mean vs. baseline. The generally significant effect was also found on a participant-by-participant basis, despite performance scatter, as shown in Figs. 4 and 5. For example, 53 out of 55 participants showed more accurate performance (greater chance of choosing the SEEN image) for SEEN = mean than for NEW = mean, when averaging results across features and presentation modes, as shown in Fig. 5, right.

We now look at the absolute distances from baseline performance for SEEN equals mean and NEW equals mean, both in the bar graphs of Fig. 3 and the scatter plots of Fig. 4. Implicit membership task performance (reporting the SEEN test image was present) is about 0.5 for baseline, better than baseline (~0.6) for SEEN = mean, and worse than baseline (~0.4) for NEW equals mean. Note, however, that the absolute difference from baseline (|0.6–0.5| and |0.4–0.5|) are closely equal and opposite. For all stimulus variables and presentation modes, the absolute difference of task performance accuracy from baseline trials (fraction selecting SEEN image) between trials where the SEEN image versus where the NEW image equals the mean, was not significant (two-tailed *t* test, *p* = 0.28–0.98). This is what would be expected if participants basically lack knowledge of image membership, and they respond only on the basis of which test image is equal to the mean.

**Fig. 6:**
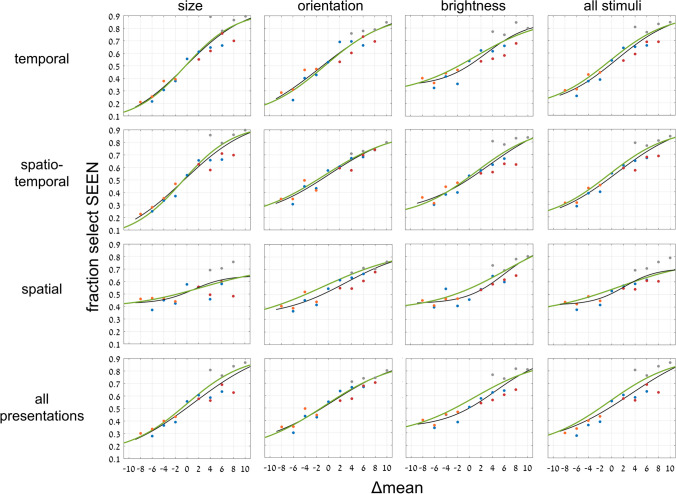
Experiment 1—*implicit, 2-test image* paradigm—Membership task performance as a function of parameter Δ for three test variables and three presentation modes. Graphs show data and best-fit sigmoid function, including data for NEW out of set range. Δ is the difference between absolute distances of NEW and SEEN images from the mean, with SEEN closer to mean on the right side of each graph, NEW closer on the left. Choice of test image closer to the mean reflects implicit ensemble perception. Red, orange, blue and gray data points reflect average performance for trials where, respectively, SEEN = mean, NEW = mean, neither = mean (baseline), and NEW is outside the ensemble range. Green curves are integral of data from Fig. 7. (Color figure online)

To include intermediate data in judging implicit mean perception—that is, not just the cases where the SEEN or NEW test image equals set mean, we introduce a new parameter, Δ. For each pair of test images, we measure the absolute distance of each test image from the mean of the set. Then we take the difference between these distances, the absolute distance of the NEW image from the mean, less the absolute distance of the SEEN image from the mean, and call this difference Δ (see examples in Fig. 2). We then plot the fraction of selecting the SEEN image as a function of Δ. As shown in Fig. 6, the result is a sigmoidal curve crossing 0 (SEEN and NEW test images equidistant from the mean) near accuracy = 0.5, that is, chance performance. This, too, is true for all variables (size, orientation, and brightness) and presentation modes (temporal, spatio-temporal, spatial). Sigmoid curves (black) in Fig. 6 are best fits to the function, $y=\mathit{min}+\frac{(max-min)}{1+{e}^{\left(-slope*\left(x-c\right)\right)}}$, with parameter ranges: min = 0 – 0.4; max = 0.64 – 1.0; c = -1.3 – 5.9; slope = 0.14 – 0.36/unit. The slopes for these data are presented in Table 3.

**Fig. 7:**
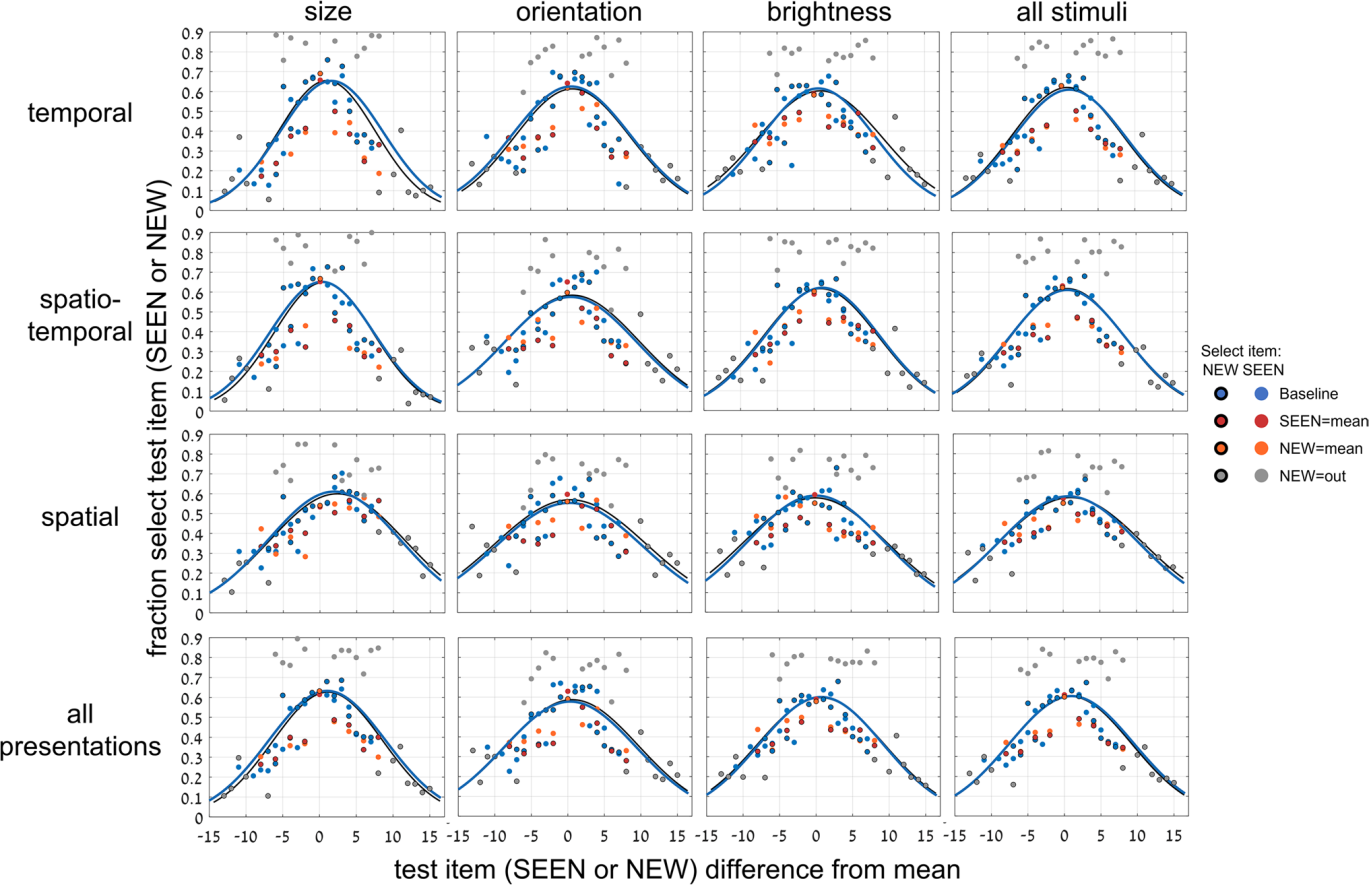
Experiment 1—*implicit, 2-test image* paradigm—Fraction responding “member of set” as a function of distance of chosen test image, whether SEEN or NEW, from set mean. Data for 3 variables and 3 presentation modes, and averages over variables, presentations, and both. Each graph shows data and best-fit Gaussian function, including data of trials with NEW out of set range. Choice of the test image closer to the mean reflects implicit ensemble perception. Framed and nonframed circles correspond respectively to fraction of selecting the NEW or the SEEN image. Blue curves are derivative of black curves of Fig. 6, where *x*-axis is not distance from mean but difference of distances from mean (Δ). (Color figure online)

Another important aspect of ensemble mean perception is the degree of precision of the percept. How precise or how broad is the representation of the mean of the set. This important aspect of ensemble perception has been dealt with previously only rarely (e.g., Hansmann‑Roth et al., 2021). To measure precision, we plot the fraction of participant responses of test image presence in the set as a function of the distance of the test image from the mean. Since this was a 2-AFC test, participants needed to choose one of the two test images, which they did by judging which was closer to the mean (as shown already in Fig. 6). If ensemble perception were just “equal or not equal to the mean,” then responses should drop to 50% when the chosen test image is not equal to the mean. Figure 7 demonstrates that this is not the case. We plot the rate of choosing a test image (whether SEEN or NEW) as a function of its distance from the mean. There is a gradual, Gaussian-curve-like decay from the peak at the point of test image equal to the mean. The width (standard deviation) of the best-fit Gaussian curve is a measure of precision of the representation of set mean. Table 2 presents σ, the Gaussian curve standard deviation (*SD*) for the averages over variable and/or over presentation mode.

**Table 2:** Experiment 1: Comparing widths of best fit implicit Gaussian (left) and Gaussian derived from implicit sigmoid (right)

Presentation:	temporal	spatio-temporal	spatial	all presentations
σ (*SD*)	7.61 / 7.25	7.77 / 7.92	9.86 / 9.49	8.35 / 8.50
Variable:	size	orientation	brightness	all variables
σ (*SD*)	7.51 / 7.95	9.04 / 8.98	8.69 / 8.46	8.35 / 8.50

Width (σ) standard deviation of best-fit Gaussian curves for each presentation mode and stimulus variable in the implicit task of Experiment 1. Left/right data reflect Gaussian of implicit choice of test image (Fig. 7, black) / Gaussian derived from implicit sigmoid of membership task performance as function of Δ (blue)

There is, of course, a mathematical connection between Gaussian and sigmoid curves. The Gaussian is just the derivative of the sigmoid and the sigmoid the integral of the Gaussian; (in both cases appropriately normalized). If the sigmoid curves in Fig. 6 (performance as a function of Δ, difference of distances from mean) reflect the same ensemble mean perception mechanism as the Gaussian curves of Fig. 7 (choice of test image as function of distance from mean), then the derived curves from each should match the other. This is indeed the case, as follows: The green sigmoid curves in Fig. 6 are the integrals of the corresponding (black) data Gaussian curves in Fig. 7 and the blue Gaussian curves in Fig. 7 are the derivatives of the corresponding (black) data sigmoid curves of Fig. 6. There is a close resemblance in all cases. Table 2 compares the Gaussian curve standard deviations for these data, and Table 3 compares the sigmoid slopes.

**Table 3:** Experiment 1: Comparing best fit slopes of implicit sigmoid curve (left) and that derived by integral of implicit Gaussian (right)

Presentation:	temporal	spatio-temporal	spatial	all presentations
Slope at midpoint	0.19 / 0.19	0.17 / 0.19	0.36 / 0.15	0.15 / 0.17
Variable:	size	orientation	brightness	all variables
Slope at midpoint	0.14 / 0.2	0.16 / 0.16	0.22 / 0.17	0.15 / 0.17

Slope of best-fit sigmoid curves for each presentation mode and stimulus variable in the implicit task of Experiment 1. Left/right data reflect sigmoid of implicit choice of test image closer to mean (Fig. 6, black) / sigmoid derived from implicit Gaussian curves of choice of test image (green)

### Experiment 1: *2-test images-explicit* test paradigm

**Fig. 8:**
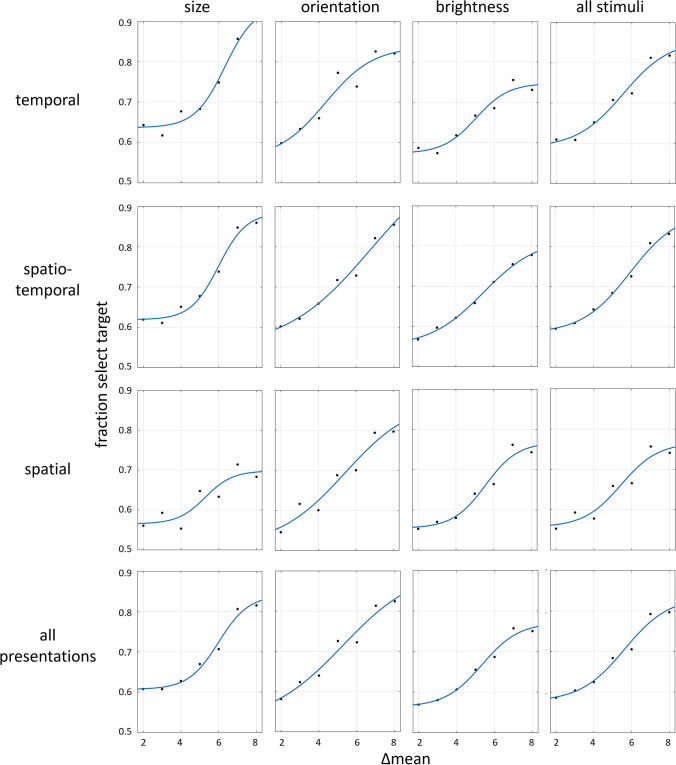
Results for Experiment 1—*Explicit, 2-test image* paradigm. Sigmoid curves of mean estimation accuracy performance as function of the relative distance of the test images (target and distractor, closer and further) from the trial mean (i.e., Δmean). A gradual increase in task accuracy is seen as a function of the difference of the test image distances from the mean, for all stimulus variables and presentation modes

Following 3 sessions testing implicit ensemble perception, we now tested explicit ensemble perception. Participants were asked, for the first time, to evaluate the mean of the set of images, and then judge which of two test images was closer to the set mean in terms of size, orientation or brightness. We expect participants to be accurate when the difference in distance from the mean for the two test images is large, and that they be less accurate when the distances are similar. Indeed, results follow a sigmoid curve, as shown in Fig. 8. Note that here the choice is between the test image that is closer to the mean versus that which is further from the mean. Table 5 (right values) presents the slopes of these best-fit curves at midpoint.

A one-way repeated-measure ANOVA (46 participants) with slope as dependent variable and 3 stimulus variables, size, orientation and brightness, as independent variable (averaged across presentation modes), showed no significant difference, *F*(2, 90) = 0.39, *p* = 0.6. The one-way repeated-measure ANOVA (44 participants) with the 3 presentation modes, temporal, spatial, and spatio-temporal, as independent variable (averaged across stimulus variables), also showed no significant effect, *F*(2, 86) = 0.79, *p* = 0.45.

### Experiment 1: *2-test images*—Comparing implicit and explicit perception

**Fig. 9:**
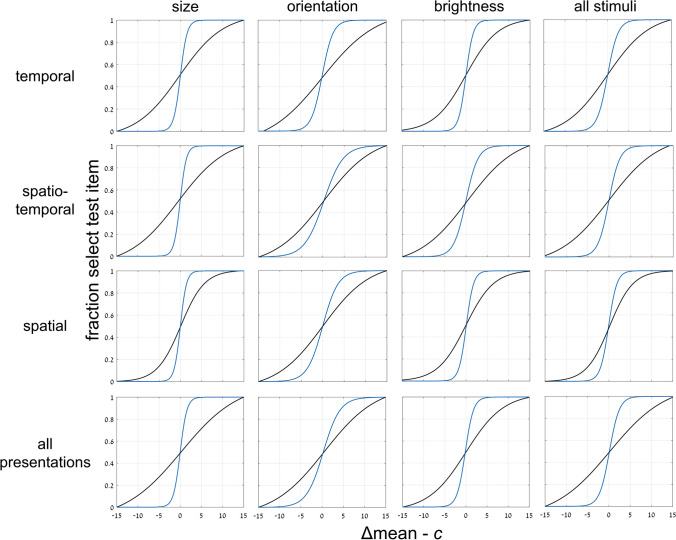
Experiment 1—*2-test images* paradigm—Comparing *implicit and explicit* ensemble perception. Normalized data for each set variable and presentation mode, and their averages. Implicit test data (black) from Fig. 6: participants asked which test image was a member of the previously presented set; Δ is the difference between distances of NEW and SEEN images from the mean. Explicit test data (blue): participants asked to explicitly estimate set mean and judge which of 2 test images is closer to the set mean; normalized data from Fig. 8, showing normalized fraction of responding to the closer test image as a function of variable Δ, the difference in distances of the test images from the mean. The explicit (blue) sigmoid has a sharper slope, compared to that of the implicit (black) sigmoid. (Color figure online)

To compare implicit and explicit ensemble perception, we plot in Fig. 9 the normalized sigmoid curves of implicit perception (black) from Fig. 6, and the normalized explicit sigmoid curves (blue) from Fig. 8. Comparing these curves, and in particular the slopes at the center (c), we see that the sigmoid curves for explicit perception (blue) are significantly steeper than those for implicit membership task performance (black); (within-subject data, averaged across presentation and stimulus types: *t* test, *p* < .001; effect size, Cohen’s *d* = 0.97). The slopes at midpoint for these curves are compared in Table 5, left versus right values for implicit versus explicit data, respectively. Note the large discrepancies between the explicit and implicit values, reflecting the sharper slopes and more precise ensemble perception for explicit tests.

**Fig. 10:**
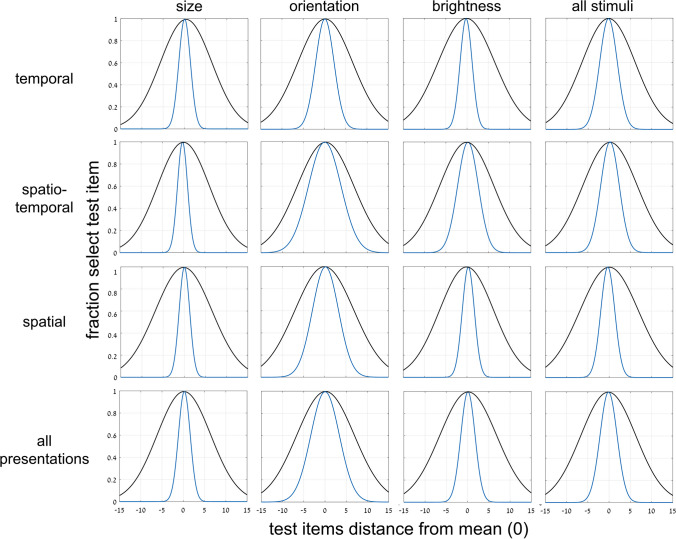
Experiment 1—comparing *implicit and explicit* ensemble perception*, 2-test images* paradigm. Normalized data for each set variable and presentation mode, and their averages. Implicit test data (black) from graphs of Fig. 7: fraction of choosing the test image as a function of its distance from the mean. Explicit test data (blue): Gaussian curve derived by taking the derivative of the (blue) sigmoid curve of Fig. 9 as a function of the difference, Δ, of the test image distances from the mean. Note the narrower Gaussian explicit (blue) curve, compared to the width of the implicit (black) Gaussian. (Color figure online)

A similar comparison can be made for the Gaussian curves for implicit perception from Fig. 7, and the Gaussian curves that can be derived by taking the derivatives of the explicit sigmoid curves of Fig. 9, as shown in Fig. 10 (implicit: black; derived from explicit: blue). Again, the explicit perception curves are narrower than the implicit perception curves, suggesting that explicit ensemble perception is more precise than implicit perception. The best-fit Gaussian curve widths are compared in Table 4, left and right values for implicit versus explicit data, respectively. Note the large discrepancies between the explicit and implicit values, reflecting the narrower curves and more precise ensemble perception for explicit tests (Table 5).

**Table 4:** Experiment 1: Comparing widths of best fit implicit Gaussian (left) and Gaussian derived from explicit sigmoid (right)

Presentation:	temporal	spatio-temporal	spatial	all presentations
σ (*SD*)	7.61 / 1.98	7.77 / 2.04	9.86 / 1.56	8.35 / 1.87
Variable:	size	orientation	brightness	all variables
σ (*SD*)	7.51 / 1.34	9.04 / 3.14	8.69 / 1.56	8.35 / 1.87

Width (σ) standard deviation of best-fit Gaussian curves for each presentation mode and stimulus variable in the implicit versus explicit tasks of Experiment 1. Left/right data reflect Gaussian of implicitly choosing a test image as present in the ensemble (Fig. 10, black curve) / Gaussian derived from explicit sigmoid (Fig. 10, blue curve), both as a function of distance of chosen test image from mean

**Table 5:** Experiment 1: Comparing slopes of best fit implicit sigmoid (left) and explicit sigmoid (right)

Presentation:	temporal	spatio-temporal	spatial	all presentations
Slope at midpoint	0.19 / 0.80	0.17 / 0.78	0.10 / 1.02	0.15 / 0.85
Variable:	size	orientation	brightness	all variables
Slope at midpoint	0.14 / 1.25	0.16 / 0.50	0.22 / 1.02	0.15 / 0.85

Slope of best-fit sigmoid curves for each presentation mode and stimulus variable in the implicit versus explicit tasks of Experiment 1. Left/right data reflect sigmoid of implicitly choosing the SEEN test image as present in the ensemble (Fig. 9, black curve) / sigmoid of explicitly choosing the Target test image as closer to the mean (Fig. 9, blue curve), both as function of relative distance of test images from mean

### Experiment 2: *1-test image—*Implicit test paradigm

**Fig. 11:**
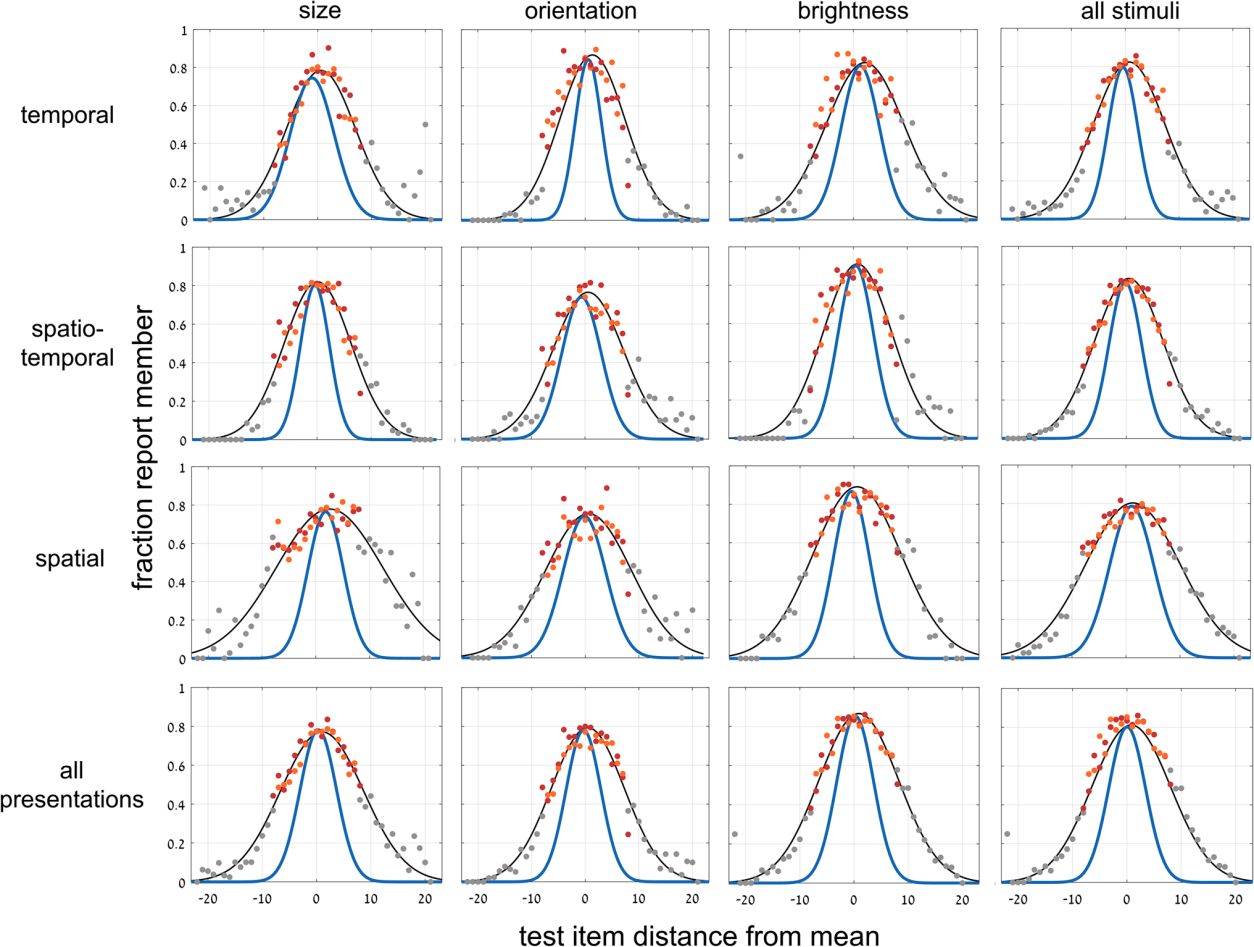
Experiment 2—*implicit, 1-test image* paradigm—Fraction responding “member of set” as a function of distance of single test image from set mean. Columns: data for 3 variables: circle size, line orientation, disc brightness, and their average; Rows: data for 3 presentation modes, temporal, spatio-temporal spatial, and their average. Graphs show data and best-fit Gaussian function (black), including data for test images included (red) or excluded (orange) from the set, or out of set range (gray). Attributing membership on basis of test image proximity to set mean reflects implicit ensemble perception. Blue curves are derivatives of corresponding sigmoid curves (of the explicit task; Fig. 12). (Color figure online)

We move now to the second experiment where the implicit or explicit tests were performed with a single test image. As shown below, the implicit-to-explicit perception comparison is more direct here. A different group of (41) participants was tested here. For the first 3 implicit sessions, participants were asked to judge if the test image had been included in the set (see Methods and Fig. 1, Top, b; we present the results of the second 3 explicit sessions below). As in Experiment 1, for the implicit sessions, we assume that it is difficult for participants to judge set membership for our brief presentation and random spacing of set members within the set range. Thus, as found above and previously (Khayat & Hochstein, 2018, 2019; and Experiment 1), participants judge membership by test image proximity to the set mean. We find a trial-by-trial Gaussian dependence of membership report on test image distance from set mean, as demonstrated in Fig. 11, for the different variables (size, orientation, brightness) and different presentation modes (temporal, spatio-temporal, spatial), and their averages.

Figure 11 includes data for cases when the test image was included in the set (SEEN image; red symbols) and when not in the set (NEW; orange). The finding that there is no difference between these cases, reflects participant lack of knowledge concerning individual set images (*t* test, *p* > .2). Data are also shown for the cases when the test image was outside the range of the set (gray), where the very low probability of responding “set member” indicates that participants perceive set range and reject outsiders. Table 6 shows σ (standard deviations, *SD*) of Gaussian curves of Fig. 11.

**Table 6:** Experiment 2: Comparing widths of best fit implicit Gaussian (left) and Gaussian derived from explicit sigmoid (right)

Presentation:	temporal	spatio-temporal	spatial	all presentations
σ (*SD*)	6.05 / 2.76	6.37 / 3.02	9.54 / 4.11	7.04 /3.38
Variable:	size	orientation	brightness	all variables
σ (*SD*)	7.05 / 3.36	6.66 / 3.25	7.24 / 3.41	7.04 /3.38

Width (σ) standard deviation of best-fit Gaussian curves for each presentation mode and stimulus variable in the implicit versus explicit tasks of Experiment 2. Left/right data reflect Gaussian of implicitly judging test image was present in the ensemble (Fig. 11, black) / Gaussian derived from explicit sigmoid (Fig. 11, blue), both as a function of distance of test image from mean

Results separately for each participant per presentation mode and stimulus variable are quite noisy, and it was not possible to fit Gaussian curves in all cases. We therefore averaged over results for all presentation modes or for all stimulus variables for computing ANOVAs, where possible. The one-way repeated-measures ANOVA (for 21 participants) with σ as the dependent variable and the 3 stimulus variables, size, orientation and brightness, as independent variable (averaged across presentation modes), showed no significant difference, *F*(2, 40) = 2.66, *p* = .082. The one-way repeated-measures ANOVA (24 participants), with the 3 presentation modes, temporal, spatial and spatio-temporal, as independent variable (averaged across stimulus variables), showed a somewhat significant effect, *F*(2, 46) = 6.75,* p* < .01. Post hoc, Type 2 *t* tests showed significant differences for spatial versus either of the other presentation modes (*p* < .05), and nonsignificant difference between temporal and spatio-temporal presentations.

### Experiment 2: *1-test image-explicit* test paradigm

**Fig. 12:**
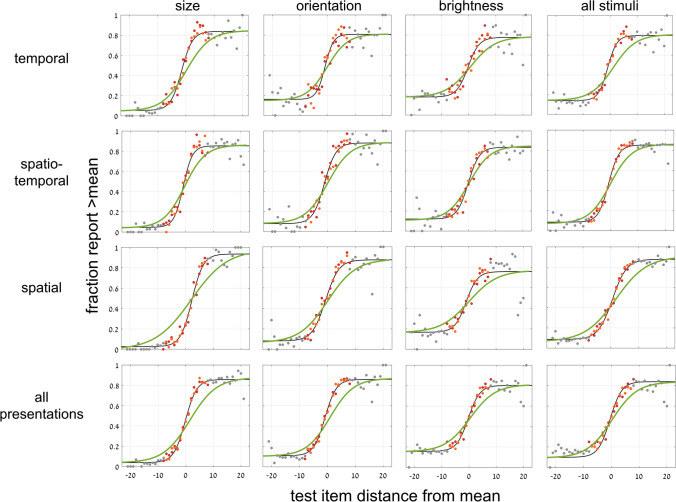
Experiment 2—*explicit, 1-test image* paradigm—Participants were explicitly asked to estimate set mean and compare it to the test image. Fraction of responding “greater” (larger, more clockwise, brighter) as a function of test image size/orientation/brightness compared to set mean. Columns: data for 3 variables. Rows: data 3 presentation modes. Graphs show data and best-fit sigmoid (black), including data for test images included (red) or excluded (orange) from the set, or out of set range (gray). Green curves are integrals of corresponding Gaussian curves of the implicit task (Fig. 11). (Color figure online)

As we did for Experiment 1, for the second part of Experiment 2 with one test image, following the 3 sessions testing implicit ensemble perception, we now tested explicit ensemble perception. Participants were directly asked to evaluate the mean of the set of images, and then to judge if the presented test image was greater than the set mean—that is, if the test circle was larger than the mean size of the set, if the test line orientation was more clockwise that the set mean orientation, or if the test disc was brighter than the set mean brightness. We expect participants to be accurate when the test image is much greater (larger, more clockwise, brighter, leading to 100% positive responses) or much less (smaller, more counterclockwise, or less bright, leading to 0% positive responses). When the test image equals or is close to the mean, responses should be close to 50% chance (or reflect response biases), and intermediate test cases should follow a sigmoidal curve. This was indeed the case, as displayed in Fig. 12, showing results for the different presentation modes and perceptual variables. There is no difference between data for test images included (red symbols) or excluded (orange) from the set (*t* test *p* > .4). Test images beyond the ensemble range (gray) are close to perfect performance, close to zero, if much smaller, and to 1, if much larger than the range. Table 7 presents values of the parameters of the best fit sigmoid curves for displays of Fig. 12.

**Table 7:** Experiment 2: Comparing best fit slopes of sigmoid derived by implicit Gaussian (left) and explicit sigmoid (right)

Presentation:	temporal	spatio-temporal	spatial	all presentations
Slope at midpoint	0.23/ 0.58	0.26/ 0.53	0.18/ 0.39	0.22/ 0.47
Variable:	size	orientation	brightness	all variables
Slope at midpoint	0.21/ 0.48	0.24/ 0.49	0.22/ 0.47	0.22/ 0.47

Slope of best-fit sigmoid curves for each presentation mode and stimulus variable in the implicit versus explicit tasks of Experiment 2. Left/right data reflect sigmoid derived from integrals of the implicit Gaussian curves of judging test image presence in the ensemble  (Fig. 12, green) / sigmoid of explicitly responding “greater than mean”  (Fig. 12, black), as a function of distance of test image from mean

A one-way repeated-measures ANOVA (25 participants) with slope as dependent variable and 3 stimulus variables, size, orientation and brightness as independent variable (averaged across presentation modes), showed no significant difference, *F*(2, 48) = 0.053, *p* = 0.9. The one-way repeated-measures ANOVA (26 participants) with 3 presentation modes, temporal, spatial and spatio-temporal, as independent variable (averaged across stimulus variables), also showed no significant effect, *F*(2, 50) = 2.66, *p* = .08. Thus, we conclude that there is little difference, if any, between performance of the tasks for different stimulus variables or for different modes of presentation.

### Experiment 2: *1-test image*—Comparing implicit and explicit perception

We now compare the results for implicit and explicit mean perception. Do they depend on the same neural computation leading to identical performance or is performance different opening the possibility that they may depend on separate mechanisms? Having found that implicit membership-test mean perception follows a Gaussian dependence on distance of the test image from the set mean (Fig. 11), and the explicit mean perception follows a sigmoidal dependence on distance of the test image from the set mean (Fig. 12), we directly compare the results. This is the same comparison method that we used in Figs. 6 and 7, but there we compared two, albeit different, implicit tests, finding no difference between them, while here we test implicit and explicit ensemble perception, and ask if these, too, are identical (as we did in Figs. 9 and 10).

We use the same natural connection between Gaussian and sigmoidal curves. We compute the integral of the Gaussian best fit (black) curves of each graph of Fig. 11, and plot the results as the green curves in Fig. 12. Similarly, we computed the derivative of the sigmoid best fit (black) curves in the graphs of Fig. 12, and plot the results as the blue curves in the graphs of Fig. 11. In all cases, the green sigmoid curves in Fig. 8, derived from the implicit data have shallower slopes than the black curves which are the best fit to the explicit data (within-subject data, averaged across presentation modes and stimulus types:* t* test, *p* < .001; effect size, Cohen’s *d* = 1.45). Similarly, in all cases, the blue Gaussian curves of Fig. 11, derived from the explicit data are narrower (smaller standard deviation) than the black curves, which are the best fit to the implicit data. Tables 6 and 7 show the values of the parameters of these derived curves and compare them with those of the directly measured curves.

We conclude that explicit mean perception is more precise than implicit mean perception, in that it results in a sharper dependence on distance from the mean, seen in both the steeper sigmoid and narrower Gaussian curves. See below (Discussion and Fig. 14) where this result is summarized, comparing data averaged over all presentation modes and variables.

**Fig. 13:**
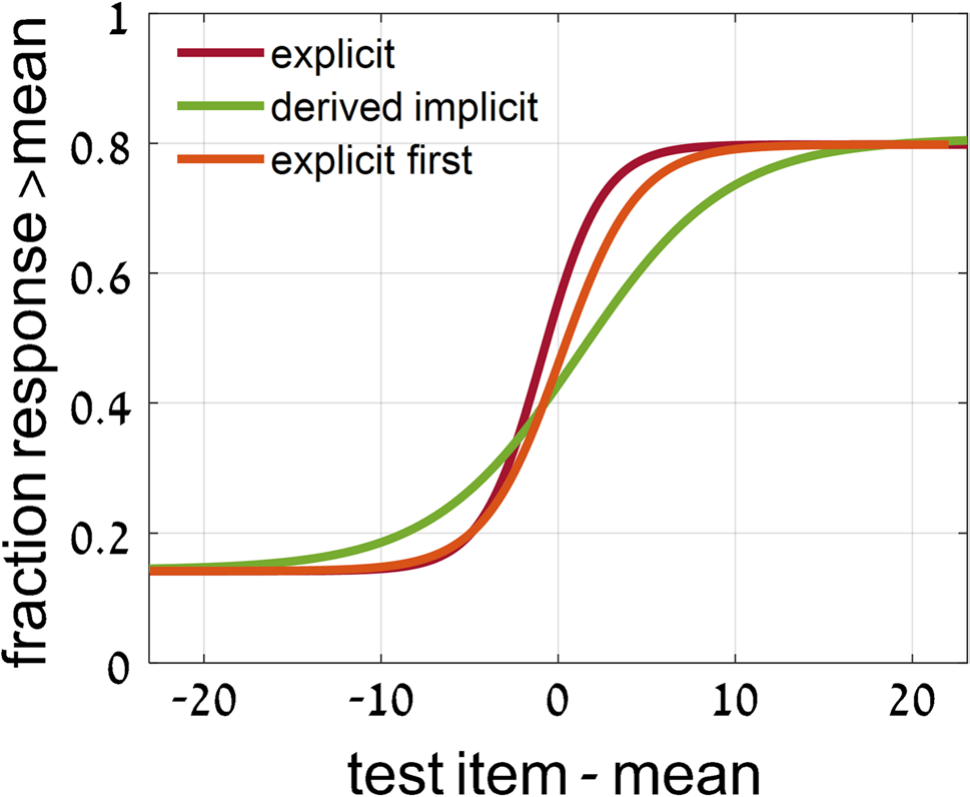
Results for Experiment 2—*implicit and explicit, 1-test image* paradigm, temporal presentation, comparing explicit and implicit ensemble perception, and introducing results for naïve participants who performed the explicit tests without prior experience with the implicit tests. Plot shows performance dependence on distance of the test image from the set mean, for the 3 variables together. The 3 curves are the best fits for the explicit test, as in Fig. 12 (red), the explicit test of the naïve participants (orange) and the sigmoid curves (of Fig. 12) derived from the implicit data of Fig. 11 (green). The orange and red explicit curves are very similar; the green implicit curve is significantly shallower, confirming that the difference between explicit and implicit ensemble performance does not derive from prior experience with the implicit tests. (Color figure online)

An objection to this conclusion may arise from the following consideration. We were careful in our experiments to first test implicit ensemble perception and only following these 3 sessions to test explicit perception. This was done to avoid participants consciously knowing that our implicit tests involve mean computation, as explicitly told them in advance of the explicit ensemble perception tests. The potential objection derives from the possibility of considerable perceptual learning being the cause of the better performance found for the explicit tests than for the implicit tests. Indeed, we have previously reported perceptual learning of ensemble perception, though there participants performed many more than 3 sessions (Hochstein & Pavlovskaya, 2020; see Hochstein et al., 2018). To rule out this potential confound, we tested a new naïve set of participants, chosen not to have had experience with any previous ensemble perception test, using the same *1-test image-explicit* test paradigm. Due to the difficulty in recruiting naïve participants, we only tested the temporal presentation mode, but still tested all three test variables. Results for the 7 naïve participants are shown in Fig. 13 (orange symbols), for the 3 variables, together. The explicit perception curves for participants tested after 3 implicit performance sessions (red) and for naïve participants tested without any prior experience are nearly identical. The curves derived from implicit performance (green) are significantly shallower.

## Discussion and conclusions

Most studies in the field of ensemble perception designed experiments with an explicit averaging task, i.e., asking participants to assess the ensemble mean. Such designs typically ask observers to adjust a test probe to reproduce the ensemble mean (e.g., Haberman et al., 2015), report on which side, or to which direction, on the feature scale, a test item is located with respect to the mean (e.g., Haberman & Whitney, 2009), or compare two sets and report which set mean is more extreme (e.g., larger/smaller, clockwise/counterclockwise, happier/sadder) in the feature scale (e.g., Chong & Treisman, 2003). Using these tasks, participants may spread their attention across the display and try to perceive a global summary percept. In contrast to this goal-driven process, implicit ensemble perception tasks are quite different, as participants do not recruit attentional resources to the global statistical properties. The effects of implicit ensemble perception are measured indirectly by their influence on some orthogonal task, such as membership tasks (e.g., Khayat & Hochstein, 2018) or visual search tasks (e.g., Chetverikov et al., 2016). Different processes may be used for explicit versus implicit task types, and it seems that at least some processing mechanisms may be unique to explicit ensemble perception such as top-down attentional strategies.

The methodology of the current study was designed to use comparable stimulus distributions and parameters (e.g., Δ) to test not only explicit and implicit mechanisms but also their use for perceiving ensembles of different features, and integration over space and time. We also employed two distinct experiments with different participants and different tasks (i.e., 1-test and 2-test image) to assess the consistency of this comparison.

We demonstrated both implicit and explicit ensemble perception for temporal, spatial and spatio-temporal presentation modes, and for ensembles with variables of circle size, line orientation, and disc brightness. In addition, we used 2 testing methodologies, with a single test image or by 2-alternative forced choice between two test images. The importance of this broad study lies first with demonstrating the ubiquitous nature of ensemble perception. Even when asked to judge whether a test image—or which of 2 test images—was present in the previously presented set of stimuli, participants always show a preference to respond according to the proximity of the test image(s) to the mean of the ensemble. With 2 test images, they more frequently choose the image closer to the mean, irrespective of whether that image was present in the set (Figs. 3, 4, 5, 6 and 7), and with 1 test image, the frequency of reporting presence in the set depends on the proximity of the test image to the set mean (Fig. 11). In both cases, choice is a Gaussian function of the distance from the mean, as demonstrated in Figs. 7 and 11, respectively. Furthermore, when presented with a 2-AFC test asking which test image was present in the set, participants choose the image that was closer to the mean, with a sigmoid dependence on the difference in distances of the test images from the mean, as shown in Fig. 6. Though tested in very different ways, these dependences of implicit perception of the ensemble mean on distance(s) from the mean are similar so that the integral of the Gaussian (of Fig. 7) matches the sigmoid (of Fig. 6), and the derivative of the sigmoid (of Fig. 6) matches the Gaussian (of Fig. 7). Similarly, the red and orange data points in Figs. 11 and 12, reflecting choice of images that were present or absent from the set, are along the same Gaussian and sigmoid curves.

**Fig. 14:**
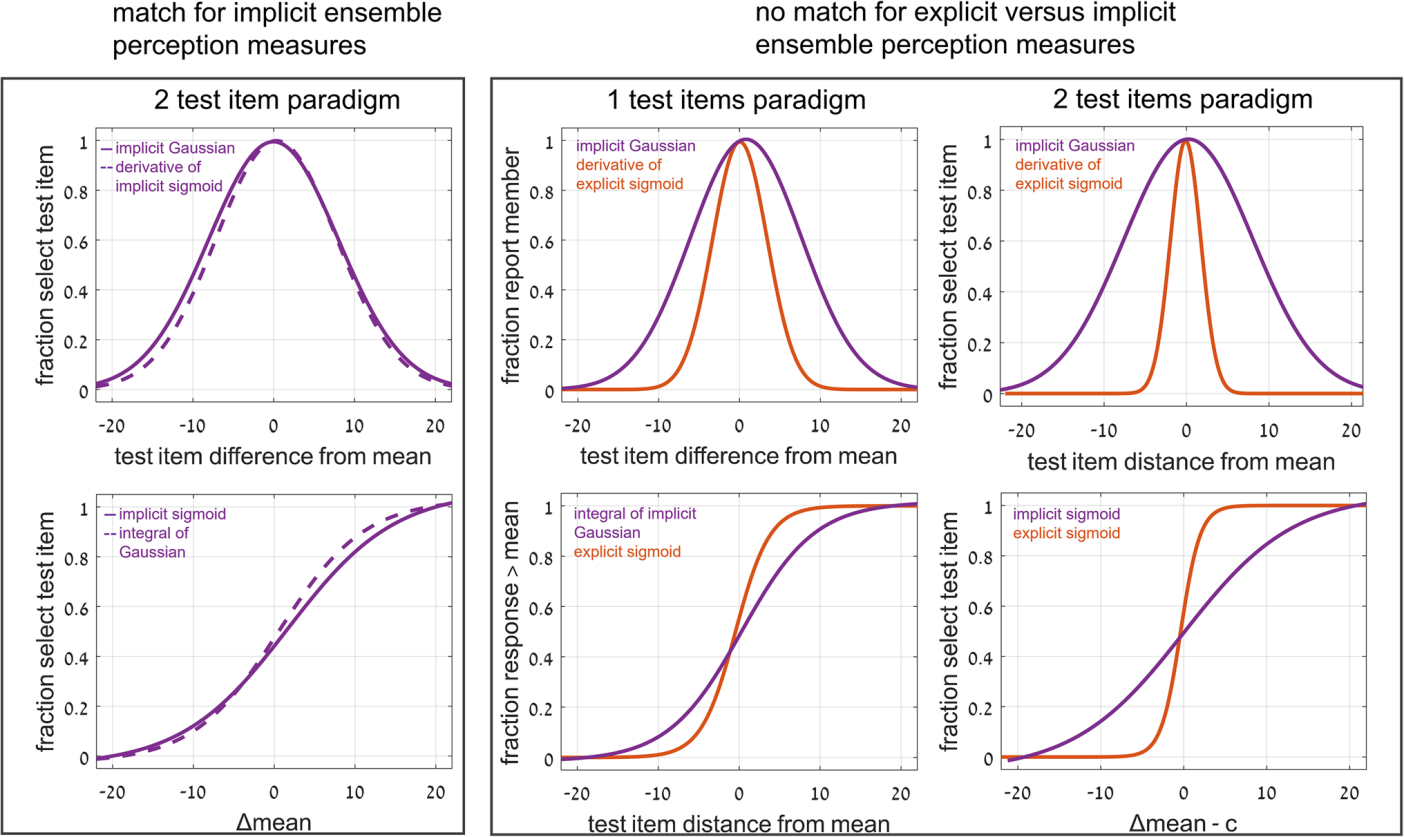
Summary of comparisons of results for different tests and measures, comparing **Top**: direct-result implicit Gaussian (purple) with derivative of either implicit (dashed purple) or explicit (orange) sigmoid; **Bottom**: direct-result implicit (purple) or explicit (orange) sigmoid curves, with curves derived by integral of implicit Gaussian (dashed purple). Averaged data over presentation modes and set variables, all normalized for comparison. **Left:** Experiment 1: *2-test image* paradigm—Comparing different tests of *implicit* ensemble perception. **Top:** Gaussian curves from Fig. 7; **Bottom:** sigmoid curves from Fig. 6. Note good coincidence of original and derived curves. **Center** (Experiment 2: 1-test image paradigm) and **Right** (Experiment 1: 2-test image paradigm)**:** Comparing *implicit and explicit* ensemble perception. **Top:** Implicit test: Gaussian curves (purple), fraction responding “member of set” as function of test image distance from set mean. Explicit test: Gaussian curves (orange) derived from sigmoid curves; from Figs. 10 and 11; *Z*-score normalization was done for all data. **Bottom: Center:** Experiment 2: *1-test image* paradigm—Explicit test: sigmoid curve (orange), fraction responding “greater” (larger, more clockwise, brighter) as function of test image size/orientation/brightness compared to sequence mean; participants asked to compare test image to explicitly estimated set mean. Implicit test: sigmoid curve (purple) derived from Gaussian curve of implicit test; from Fig. 12. **Right:** Experiment 1: *2-test image* paradigm—Explicit test: sigmoid curve (orange), fraction explicitly choosing test image closer to mean as function of difference of test images’ distances from sequence mean. Implicit test: sigmoid curve (purple) of implicit test; from Fig. 9. Note lack of coincidence of original and derived curves. Narrower Gaussians and steeper sigmoid slopes for explicit data indicate explicit ensemble perception is more precise. (Color figure online)

Importantly, this type of curve superposition was not found when comparing performance of these implicit ensemble perception tests with direct explicit perception tests. Only following the implicit tests, participants were informed that they would now be tested on perception of the ensemble mean. With 2 test images, they were asked to judge which was closer to the mean, and with 1 test image, they were asked to judge if it was greater than the mean—that is, larger, more clockwise, or brighter that the set mean circle, line or disc. With 2 test images, explicit ensemble perception is reflected in a sigmoid dependence of choosing an image as closer to the mean on the difference between the distances of the two test images from the mean (Figs. 8 and 9). Note that this is dependence on the same parameter Δ, the difference in distances of the two test images from the mean, as used for the implicit 2-image ensemble perception test (Fig. 6), though here we test explicit choice of the image that is closer to the mean, rather than implicit use of Δ to choose images closer to the mean. With 1 test image, there is also a sigmoid dependence on the distance from the mean (Fig. 12): When the test image is much larger, more clockwise, brighter than the mean, participants nearly always report “greater than the mean,” and when much smaller, more counterclockwise, dimmer, they almost never report “greater,” with a sigmoid dependence between these extremes (Fig. 12). The important result is that when comparing these sigmoid curves with the implicit results, the two are not equivalent. Instead, explicit perception has a steeper sigmoid, and narrower Gaussian, as demonstrated in Figs. 11 and 12, for 1 test image, and Figs. 9 and 10, for 2 test images. Figure 14 summarizes the results of these tests and comparisons, showing equivalence of difference implicit tests (left column) and the lack of equivalence when comparing explicit and implicit tests of ensemble perception (central and right columns for 1 and 2 test images, respectively).

One of the goals of this broad comparative study was to seek evidence concerning the relationships among the mechanisms underlying different tasks. Is there a single “averaging” mechanism that performs mean perception for ensembles differing in various features, and/or spread over space or time, and when observers perform an averaging task or implicitly perceive the mean when engaged in an unrelated task, or are there separate cerebral mechanisms for some or all of these different tasks? Taking the above results together, a possible conclusion would be that the 3 stimulus variables and 3 presentation modes all use the same underlying mechanism(s) for computing the mean, since the results are so similar for all 9 tests (and for the 2 testing methodologies). The slight differences for spatial presentation might suggest a different mechanism for this mode. In contrast to these similarities, the significant difference in results for explicit and implicit ensemble perception might suggest that difference mechanisms underly these phenomena.

Interestingly, another recent discrimination was reported between explicit and implicit ensemble perception. Hansmann-Roth et al. (2021) report that conscious awareness appears to have access only to basic summary statistics (e.g., mean and variance), but the entire feature distribution has only implicit effects on behavior.

Nevertheless, we hesitate to conclude that this difference in precision necessarily reflects different underlying mechanisms. It is possible, as well, that the same mechanism is responsible for both implicit and explicit ensemble perception, but that this mechanism is used more efficiently, or depends on more reliable information, when attention is paid to the stimuli and their mean explicitly. Ultimately, resolving this issue of one or more mechanisms may depend on analysis of individual differences in these tests. We are now performing just such an analysis.
